# ExplorEnz: a MySQL database of the IUBMB enzyme nomenclature

**DOI:** 10.1186/1471-2091-8-14

**Published:** 2007-07-27

**Authors:** Andrew G McDonald, Sinéad Boyce, Gerard P Moss, Henry BF Dixon, Keith F Tipton

**Affiliations:** 1 School of Biochemistry and Immunology, Trinity College, Dublin 2, Ireland; 2 Department of Chemistry, Queen Mary University of London, London, UK; 3 King's College, Cambridge, UK

## Abstract

**Background:**

We describe the database ExplorEnz, which is the primary repository for EC numbers and enzyme data that are being curated on behalf of the IUBMB. The enzyme nomenclature is incorporated into many other resources, including the ExPASy-ENZYME, BRENDA and KEGG bioinformatics databases.

**Description:**

The data, which are stored in a MySQL database, preserve the formatting of chemical and enzyme names. A simple, easy to use, web-based query interface is provided, along with an advanced search engine for more complex queries. The database is publicly available at . The data are available for download as SQL and XML files via FTP.

**Conclusion:**

ExplorEnz has powerful and flexible search capabilities and provides the scientific community with the most up-to-date version of the IUBMB Enzyme List.

## Background

The Nomenclature Committee of the International Union of Biochemistry and Molecular Biology (NC-IUBMB), in association with the IUPAC-IUBMB Joint Commission on Biochemical Nomenclature (JCBN), is responsible for the classification of enzymes and production of the IUBMB Enzyme List. The NC-IUBMB assigns EC numbers to enzymes and provides a brief synopsis of each enzyme, a work that is coordinated by our group at Trinity College Dublin. These data are then used by many other resources, including the Swiss-Prot ENZYME, BRENDA and KEGG databases.

### The classification system: a brief description

When classified, each enzyme is assigned a four-part EC number, in the form of digits separated by periods. The first three numbers represent the class, subclass and sub-subclass to which an enzyme belongs, and the fourth digit is a serial number to identify the particular enzyme within a sub-subclass. The class, subclass and sub-subclass each provide additional information about the reaction classified. For example, in the case of EC 1.2.3.4, the digits indicate that the enzyme is an oxidoreductase (class 1), that it acts on the aldehyde or oxo group of donors (subclass 2), that oxygen is an acceptor (sub-subclass 3) and that it was the fourth enzyme classified in this sub-subclass (serial number 4).

In addition to the EC number, other information about the enzyme is provided so that the user can get a flavour of the enzyme's function and how it differs from similar enzymes. This additional information is divided into the following fields: accepted name, reaction, glossary, synonyms, systematic name, comments, references and links to other databases. Diagrams of individual reactions or of the related metabolic pathways are also provided in many instances. Further details of the classification system can be found elsewhere [1,2]. An important aspect of the Enzyme List is that it attempts to ensure a high degree of accuracy and quality for each enzyme entry. Thus, for example, a new enzyme is added only when there is sufficient, published, evidence that the reaction claimed is actually catalysed by a single enzyme that differs from all previously listed enzymes.

The IUBMB enzyme data are publicly available on the web [3] as a series of flat files. While a number of endeavours already use the enzyme data as an integral subset of the data they provide – for example, the BRENDA [4], ExPASy [5], GO [6], IntEnz [7] and KEGG [8] databases – the manually curated IUBMB enzyme data are not distinguished from the other data provided. In addition, the formatting of chemical names is, in many cases, not in accordance with IUBMB recommendations (e.g. no subscripts, superscripts or italicization of locants), although otherwise the names are semantically accurate. In this article, we present ExplorEnz as an alternative means of accessing the most up-to-date Enzyme Nomenclature information, in a readily searchable manner and with correctly rendered output.

## Construction and content

The enzyme data and their associated literature references are stored in MySQL databases on a dedicated server, and are accessed through a web interface written in PHP. The initial content for the database was extracted from the HTML-formatted flat files located on the home page of the IUBMB Enzyme List [3]. Custom Perl scripts were used to strip out the hard-coded HTML formatting and to convert the data into a plain ASCII flat file. A second set of Perl scripts was written to convert the plain-text data into HTML. A unique feature of these scripts is that they include rules to automatically generate the correct formatting of chemical names and formulae using a regular-expression-based pattern-matching system. This set of regular-expression-based replacement rules has been incorporated into its own database for use within this and other web applications.

**Figure 1:**
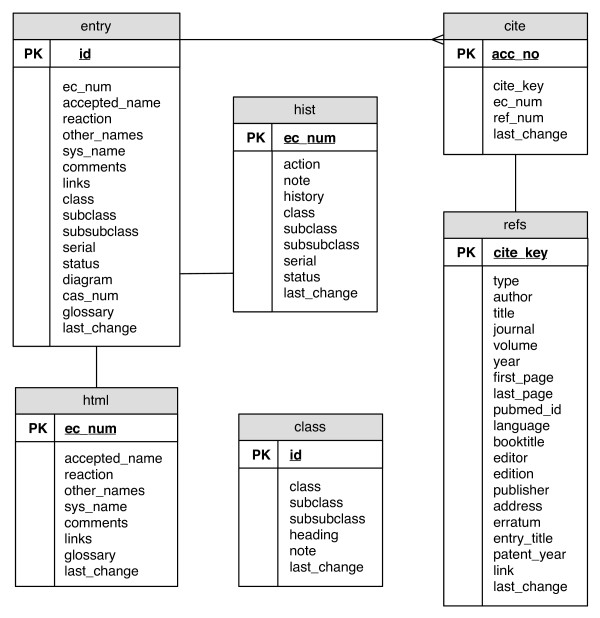
Schematic outline of the structure of the MySQL ExplorEnz database.

The arrangement of the MySQL database is shown schematically in Fig. 1. It currently comprises six tables, containing information that can be divided into two categories: enzyme data and supporting literature references. One table is used to store information on each EC class, subclass and sub-subclass; three others store the searchable data (i.e. plain-text data), the HTML data and a table in which are stored the status and history of an EC number. Literature references are assigned a unique citation key and are stored in a fifth table; the sixth table relates the citation key to an individual enzyme entry.

In addition to the public database, a curatorial interface was also developed, which provides members of the reviewing panel with real-time access to all data on new/amended enzymes in an effort to speed up the classification process. The interface allows direct entry or modification of data in individual fields as plain text, which is then automatically rendered into the correct format. References can be imported automatically into the database using PubMed (PMID) numbers. All changes to the database are logged, which enables tracking of all changes made on a specific date or to a particular enzyme entry over time. A script was also written to convert these data into the format used on the IUBMB website [3], to prevent duplication of effort and to ensure consistency among the IUBMB data sets.

## Utility and Discussion

**Figure 2:**
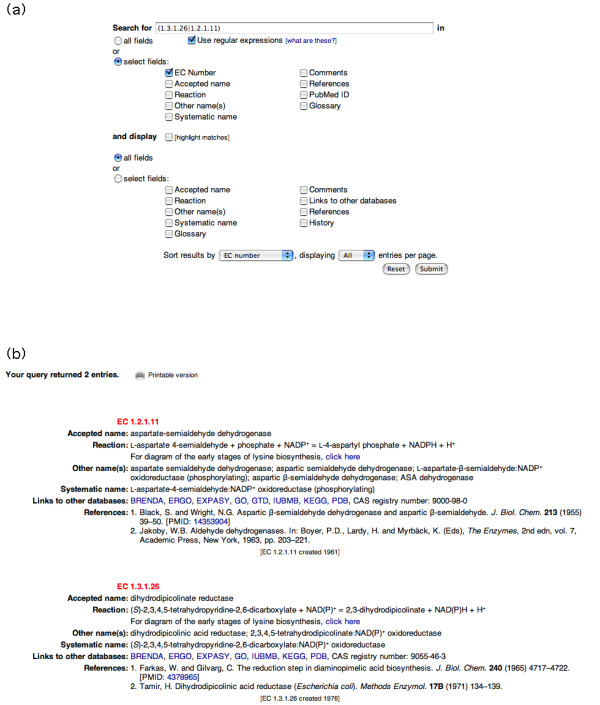
The search function of ExplorEnz. **A**. The default search interface of ExplorEnz, including the regular-expression query that provides the search results shown in **B**, where the first two of four results are shown.

The search interface provides text searching of all or a selected subset of the fields held in the database, as shown in Fig. 2(a). The wildcard character is the asterisk (*). By default, all of the fields in the enzyme entry are displayed in the results, but the user has the option to limit the output displayed to fields they select; for example, it is possible to search for all enzymes that contain the word 'glucose' within the fields Accepted name and Systematic name but to display only the contents of the Reaction field of each database record, along with the EC number. By default, all of the entries that match the search criteria are displayed on a single page. Alternatively, one can specify the number of entries to be displayed on each page (from 1 to 250, in predefined increments).

ExplorEnz makes use of the regular-expression matching facility of MySQL, thus allowing the user to construct more complex queries; since text fields within the database are set as case-insensitive, the most basic use of this feature would be for case-sensitive search functionality. In addition, there is an "Advanced search" facility that allows the user to search for up to four different text patterns at once, using Boolean algebra to include or exclude terms from the selected fields. To our knowledge, this range of search and display options is unavailable in other enzyme databases at present. Fig. 2(b) shows the result of searching for some of the enzymes involved in the early stages of lysine biosynthesis. This query takes advantage of the regular-expression-based search facility to limit the search to specific EC numbers, i.e. EC 1.3.1.26 and EC 1.2.1.11.

While the database returns its results as HTML, the user-supplied term is matched against a plain-ASCII version of the data. In the majority of cases, queries can be posed unambiguously; bold, italic, subscripted and superscripted entities should be submitted inline without any modifier: for example, either "tRNATyr" or "trnatyr" can be used to match entries that appear in the output as "tRNA^Tyr^". Greek letters should be spelt out in English: e.g., "alpha" for "α ", "beta" for "β", "delta" for "δ", "Delta" for "Δ", etc.

**Figure 3:**
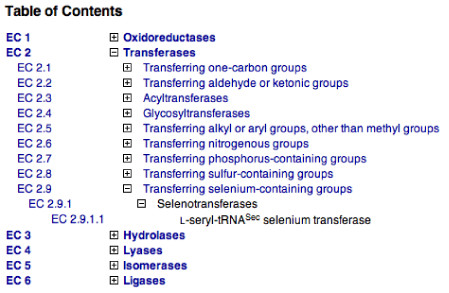
EC Table of Contents from ExplorEnz. Enzyme classes, subclasses and sub-subclasses can be expanded, to reveal their contents, or else collapsed, by clicking on the "+" or "-" symbols, respectively. Selecting a partial EC number will display the complete records of all entries in that range; clicking on a complete EC number will search for that enzyme entry alone. The class and subclass headings are linked to descriptions of their contents.

Unless the search is restricted to the EC-number field, all enzyme entries that match the search term in any (selected) field will be returned. For example, searching for "1.1.1.1" will return entries with EC numbers 1.1.1.1, 1.1.1.10, 1.1.1.11, etc., as well as those entries that contain any references to those EC numbers. Searches that are restricted to the EC-number field alone will match EC numbers exactly, unless a wildcard character is included. Alternatively, any part of the EC number can be replaced by a wildcard; hence, an EC-only search for "1.1.1.*" will return all enzyme entries with EC numbers in sub-subclass 1.1.1. Another way to search for an EC number is by using the dynamically generated table of contents of the Enzyme List. As shown in Fig. 3, the table of contents display the class, subclass, sub-subclass and accepted names of each whole or partial EC number, which, when clicked upon, will return the relevant enzyme entry, or set of entries. Clicking on the class or subclass title (e.g. "Oxidoreductases") will open a separate window with information describing the contents of that class or subclass. The data on a specific enzyme entry can be obtained by entering the EC number in the 'Look up EC number' text box at the top of the homepage or by using a special URL specifying the EC number [9].

There is also the option of outputting the results in a format that is more suitable for printing (Print Version button). In this case, the font size is reduced to make the text more compact, the output is rendered in black and white, the 'Links to other databases' field is omitted and all underlining of links is suppressed. Alternatively, the printable version can be saved as a PDF file to the user's hard disk if the user has an appropriate OS or relevant third-party software. The user's search term can be highlighted in the results page, a feature that takes advantage of the regular-expression formatting to compute the string that becomes highlighted in the HTML data. Thus, entering "alpha-D-glucose" as a search term, and with highlighting selected, will result in each occurrence of "α-D-glucose" being highlighted in the output.

The diagrams of enzyme reaction mechanisms and pathways, produced by Moss and Dixon for the Enzyme List [1,3], are also available through ExplorEnz. The diagrams show the structures of the substrates and products and, in the case of reaction mechanisms, the intermediates. EC numbers, where shown, are linked to the corresponding entries in the database. The diagrams are supplied as GIF images, although it is hoped to provide Scalable Vector Graphic (SVG) versions in the future, as this would allow the user to search for chemical names and EC numbers within the diagrams.

### Database curation and the automatic formatting of chemical names

A distinguishing characteristic of ExplorEnz is its preservation of the formatting of chemical names. Table 1 shows some examples of the formatting achieved using the regular-expression rules developed for this purpose. The unformatted names form part of the searchable data, while the formatted versions are stored within the database as HTML. The display data are pre-rendered for greater efficiency, but we have developed a curatorial interface that converts plain text entered by the curator into HTML automatically using the regular-expression database referred to earlier. The context-sensitive nature of the formatting necessitates the imposition of conditions. For example, the substitution that is used to italicize the locant 'N' in chemical names is not active on the strings "N-terminal" or "N-terminus" because of an auxiliary condition, stored in the database, which contains these as exceptions.

**Table 1:** Some examples of the formatting of enzyme and chemical names.

**Enzyme Entry**	**Field**	**Unformatted and Formatted Data**
EC 1.3.1.71	Accepted name	Delta24(241)-sterol reductase Δ^24(241)^-sterol reductase
EC 2.1.1.18	Systematic name	S-adenosyl-l-methionine:1,4-alpha-d-glucan 6-O-methyltransferase *S*-adenosyl-l-methionine:1,4-α-d-glucan 6-*O*-methyltransferase
EC 2.4.2.31	Reaction	NAD(P)+ + l-arginine = nicotinamide + Nomega-(ADP-d-ribosyl)-L-arginine NAD(P)^+ ^+ l-arginine = nicotinamide + *N*^ω^-(ADP-d-ribosyl)-l-arginine
EC 5.1.3.20	Systematic name	ADP-l-glycero-d-manno-heptose 6-epimerase ADP-l-*glycero*-d-*manno*-heptose 6-epimerase
EC 5.3.3.13	Other name(s)	eicosapentaenoate cis-Delta5,17-eicosapentaenoate cis-Delta5-trans-Delta7,9-cis-Delta14,17 isomerase eicosapentaenoate *cis*-Δ^5,17^-eicosapentaenoate *cis*-Δ^5^-*trans*-Δ^7,9^-*cis*-Δ^14,17 ^isomerase

Such conditions can readily be converted, on retrieval, to the regular-expression syntax of the language in which the web application is written. This feature reduces the time required for the curator to input data and ensures consistency of formatting throughout the database. The direct output of the data in IUBMB nomenclature format should be of benefit to journal editors wishing to check standardized usage, and the comprehensive searching facility, including searches by synonyms or reactants, should facilitate the ready identification of novel enzymes that should be included in the Enzyme List.

At the time of writing (June 2007), ExplorEnz holds 1108 class-1 entries (oxidoreductases), 1162 class-2 entries (transferases), 1111 class-3 entries (hydrolases); 356 class-4 entries (lyases); 160 class-5 entries (isomerases) and 139 class-6 entries (ligases): a total of 4036 enzymes. EC numbers no longer in use are listed as being either deleted or transferred, of which there are 784 instances, giving a total of 4809 EC numbers. A more detailed version of these data is given in Table 2.

**Table 2:** Statistics on EC numbers held in the database.

	Class 1 (Oxidoreductases)	Class 2 (Transferases)	Class 3 (Hydrolases)	Class 4 (Lyases)	Class 5 (Isomerases)	Class 6 (Ligases)	All classes
Current	1,108	1,162	1,111	356	160	139	4,036
Transferred	146	48	276	63	3	1	537
Deleted	60	57	98	21	7	4	248
Total	1,253	1,208	1,382	416	163	140	4,821

## Conclusion

A key attribute of ExplorEnz is its superior search and display functionality. Data in the HTML output are formatted according to accepted conventions, something that few databases have implemented to date. This database is the primary source of new EC numbers, from which all other databases containing the Enzyme Nomenclature data can be updated. To this end, we have made provision for MySQL replication of ExplorEnz to interested parties. In addition, daily updates of the data are made available for download in both SQL and XML format on the ExplorEnz website.

## Availability and requirements

The ExplorEnz website is publicly available at . The data are accessible as (gzip-compressed) SQL and XML files via FTP from  and . Users are requested to acknowledge the IUBMB as the source of these data.

## Authors' contributions

AM designed the database, wrote the web interface, and drafted the manuscript. SB and AM designed the web and curatorial interfaces, which were programmed by AM and tested by SB and KT, who also assisted in drafting the manuscript. GM and HD contributed pathway diagrams and enzyme mechanisms referred to in the paper and tested the web interface.
